# Bicarbonate enhances expression of the endocarditis and biofilm associated pilus locus, *ebpR-ebpABC*, in *Enterococcus faecalis*

**DOI:** 10.1186/1471-2180-10-17

**Published:** 2010-01-21

**Authors:** Agathe Bourgogne, L Charlene Thomson, Barbara E Murray

**Affiliations:** 1 Division of Infectious Diseases, Department of Medicine, University of Texas Medical School, (6431 Fannin) Houston, Texas (77030), USA; 2 Center for the Study of Emerging and Reemerging Pathogens, University of Texas Medical School, (6431 Fannin) Houston, Texas (77030), USA; 3 Department of Microbiology and Molecular Genetics, University of Texas Medical School, (6431 Fannin) Houston, Texas (77030), USA

## Abstract

**Background:**

We previously identified *ebpR*, encoding a potential member of the AtxA/Mga transcriptional regulator family, and showed that it is important for transcriptional activation of the *Enterococcus faecalis ***e**ndocarditis and **b**iofilm associated **p**ilus operon, *ebpABC*. Although *ebpR *is not absolutely essential for *ebpABC *expression (100-fold reduction), its deletion led to phenotypes similar to those of an *ebpABC *mutant such as absence of pili at the cell surface and, consequently, reduced biofilm formation. A non-piliated *ebpABC *mutant has been shown to be attenuated in a rat model of endocarditis and in a murine urinary tract infection model, indicating an important participation of the *ebpR-ebpABC *locus in virulence. However, there is no report relating to the environmental conditions that affect expression of the *ebpR-ebpABC *locus.

**Results:**

In this study, we examined the effect of CO_2_/HCO_3_^-^, pH, and the Fsr system on the *ebpR-ebpABC *locus expression. The presence of 5% CO_2_/0.1 M HCO_3_^- ^increased *ebpR-ebpABC *expression, while the Fsr system was confirmed to be a weak repressor of this locus. The mechanism by which the Fsr system repressed the *ebpR-ebpABC *locus expression appears independent of the effects of CO_2_^- ^bicarbonate. Furthermore, by using an *ebpA*::*lacZ *fusion as a reporter, we showed that addition of 0.1 M sodium bicarbonate to TSBG (buffered at pH 7.5), but not the presence of 5% CO_2_, induced *ebpA *expression in TSBG broth. In addition, using microarray analysis, we found 73 genes affected by the presence of sodium bicarbonate (abs(fold) > 2, *P *< 0.05), the majority of which belong to the PTS system and ABC transporter families. Finally, pilus production correlated with *ebpA *mRNA levels under the conditions tested.

**Conclusions:**

This study reports that the *ebp *locus expression is enhanced by the presence of bicarbonate with a consequential increase in the number of cells producing pili. Although the molecular basis of the bicarbonate effect remains unclear, the pathway is independent of the Fsr system. In conclusion, *E. faecalis *joins the growing family of pathogens that regulates virulence gene expression in response to bicarbonate and/or CO_2_.

## Background

Enterococci are part of the normal flora in human intestines and are also a leading cause of nosocomial infections [1,2]. These organisms are somehow able to migrate from the gastrointestinal tract into the bloodstream and cause systemic infections such as bacteremia and even endocarditis [2-4]. Although many strains of enterococci seem to be harmless commensals, particular subgroups of *Enterococcus faecalis *and *Enterococcus faecium *predominate among isolates from nosocomial enterococcal infections. In *E. faecalis*, numerous factors important for virulence have been characterized. For example, the Fsr system, a homologue of the staphylococcal Agr system, has been shown to be important for virulence due, at least in part, to its control of gelatinase and a serine protease expression via a quorum-sensing mechanism [5-7]. Microarray studies also indicated that the Fsr system regulates other genes important for virulence [8], one of which is the locus encoding Ebp pili [8], whose subunits are encoded by the *ebp *operon [9]. A non-piliated *ebp *mutant, producing much less biofilm than the parent strain, was shown to be attenuated in a rat model of endocarditis [9] and in a murine urinary tract infection model [10]. We previously described EbpR as an important activator of the *ebpABC *operon encoding the pili in *E. faecalis *OG1RF [11]. Although *ebpR *is not essential for *ebpABC *expression, we detected 100-fold less *ebpABC *mRNA in a Δ*ebpR *mutant compared to the OG1RF parent strain. In addition, even in the presence of an intact *ebpR *gene, only 5-20% of the cells, grown aerobically in BHI or in TSBG, were found to produce pili (detected by electron microscopy or immunofluorescence) [9,11]. These results imply that other regulatory and/or environmental factors may affect pilus production.

Bicarbonate is a major element of the mammalian body for reaching and maintaining homeostasis. In equilibrium with CO_2_, H_2_CO_2 _and CO_3_^2-^, depending on pH, temperature, and CO_2 _pressure, bicarbonate does not diffuse freely across the membrane and needs specific transporters [12]. In the stomach, HCO_3_^- ^is secreted by the surface mucus cells, where it gets trapped in the mucus and forms part of the mucus-HCO_3_^- ^barrier, thereby maintaining a pH gradient of pH 2 in the lumen to pH 7 at the mucosal epithelium interface. Interestingly, some microbial pathogens have been shown to respond in vivo to CO_2 _(from 5 to 20%) and/or HCO_3_^- ^(10-100 mM) by enhancing production of factors important for virulence (*Staphyloccocus aureus *[13], *Vibrio cholerae *[14], group A streptococcus [15], *Bacillus anthracis *[16,17], *Cryptococcus neoformans *[18] and *Citrobacter rodentium *[19]). Regulatory proteins have been described which mediate the CO_2_/HCO_3_^- ^response at the transcriptional level in *B. anthracis *(AtxA-like proteins [20]), in Group A streptococci (Mga [21]) and, recently, in *C. rodentium *with RegA [19]. For *E. faecalis*, except for a report showing an increase in cytolysin expression when grown in 80% H_2_-20% CO_2 _[22], we could find no other report of a CO_2_/HCO_3_^- ^effect on known virulence-associated genes. A candidate for such study is the *ebpABC *operon and its regulator, *ebpR*, a gene encoding a transcriptional regulator affiliated with the AtxA/Mga family; as mentioned above, this family is known to have its regulon activated in response to elevated CO_2 _[15,23].

In the present study, we report the identification of environmental conditions affecting the expression of the *ebpR-ebpABC *locus and, consequently, pilus production. In addition, we found that Fsr repressed the *ebpR-ebpABC *locus in all conditions tested, independent of the CO_2_/bicarbonate effect. Finally, among the dozens of genes that are differentially expressed after being exposed to bicarbonate, the majority belong to the PTS system and ABC transporter families.

## Results

### *ebpR *and *ebpA *expression profiles when grown aerobically in TSBG

**Figure 1:**
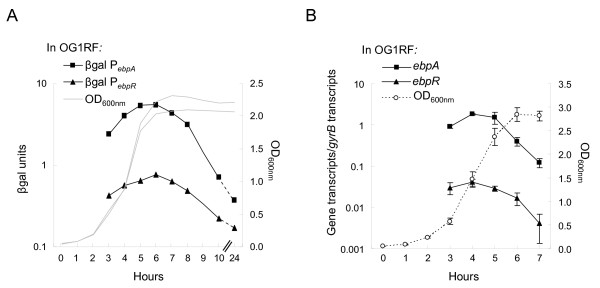
***ebpR *and *ebpA *expression profiles in OG1RF**. **A**. Expression levels of *ebpA *and *ebpR *using gene promoter::*lacZ *fusions. OG1RF containing either P_*ebpR*_::*lacZ *(black triangle) or P_*ebpA*_::*lacZ *(black square) were grown in TSBG. For β-gal assays, samples were collected every hour from 3 to 8 hr, then at 10 and 24 hr after starting the culture (x axis). The left axis represents the β-gal units (OD_420 nm_/protein concentration in mg/ml). The right axis indicates the OD_600 nm _readings. All sets of cultures presented were analyzed concurrently. This figure is a representative of at least three independent experiments. **B**. qRT-PCR with RNA purified from OG1RF cultures grown aerobically in TSBG. The left axis represents the level of transcript normalized to *gyrB *transcript level. The right axis indicates the OD_600 nm _readings. The dashed line shows the mean (with standard deviation) of 5 independent cultures of OG1RF grown in TSBG. The transcript levels of *ebpR *(black triangle) and *ebpA *(black square) shown represent two different data sets, each tested in duplicate that were normalized using *gyrB *transcript levels.

We previously identified an *E. faecalis *transcriptional regulator, EbpR, which positively affects the expression of the endocarditis and biofilm-associated pilus operon, *ebpABC *[11]. To further explore *ebpR *and *ebpABC *expression profiles, we created *lacZ *fusions with the *ebpR *and *ebpA *promoters (P_*ebpR*_::*lacZ *and P_*ebpA*_::*lacZ*). We first tested the time course of expression of *ebpR *and *ebpA *in OG1RF grown aerobically in TSBG (our standard biofilm medium) from mid-log growth phase to late stationary. In these conditions, each fusion showed the same general dome-shape pattern that reached a peak between 5 and 6 hr (Fig. 1A); specifically, the β-gal units for OG1RF carrying the *ebpA *promoter were 2.4, 5.4, and 0.4 at mid-log (3 hr after starting the culture), entry into stationary (5 hr) and late stationary growth phase (24 hr), respectively, while the *ebpR *fusion generated consistently lower β-gal units than the *ebpA *fusion.

Since β-galactosidase assays reflect translation as well as transcription, we also directly explored the steady-state mRNA levels of transcripts of *ebpR *and *ebpA *with qRT-PCR in the same conditions used above (TSBG, aerobically) compared to the housekeeping gene *gyrB*. At the peak of *ebpR *expression, which occurred between mid- and late log phase growth, the ratio between *ebpR *and *gyrB *transcript levels was 0.04 (Fig. 1B). After entry into stationary phase, *ebpR *expression decreased to an *ebpR*/*gyrB *ratio of 0.004 representing a 10-fold decrease when compared to late log growth phase levels. Likewise, *ebpA *expression also peaked at the late log growth phase with an *ebpA*/*gyrB *ratio of 1.5 and decreased to a ratio *ebpA/gyrB *of 0.12 (also a 10-fold reduction when compared to *ebpA *expression level at late log growth phase). The *ebpA *steady-state mRNA levels were an average of 37-fold higher than *ebpR *steady-state mRNA levels. Overall, the patterns between qRT-PCR and the β-gal assays were similar except for a one-hour delay for peak expression in the β-gal assays, probably due to a delay between transcription and translation.

### The CO_2_-NaHCO_3 _induction effect on *ebpR *and *ebpA *expression

**Figure 2:**
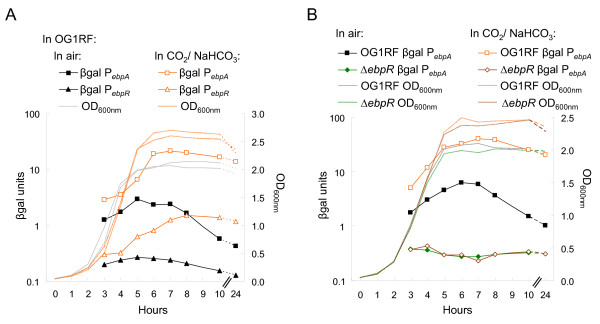
**CO_2_/NaHCO_3 _induction effect on *ebpA *expression level**. Samples were collected every hour from 3 to 8 hr, then at 10 and 24 hr after starting the culture in TSBG. The left axis represents the β-gal units (OD_420 nm_/protein concentration in mg/ml). The right axis indicates the OD_600 nm _readings. All sets of cultures presented were analyzed concurrently. Each figure is a representative of at least three experiments. **A**. Growth curves of OG1RF are shown in gray (in air) and in orange (in the presence of 5% CO_2_/0.1 M NaHCO_3_). OG1RF containing P_*ebpR*_::*lacZ *(triangle) or P_*ebpA*_::*lacZ *(square) was grown in air (closed black symbol) or in the presence of 5% CO_2_/0.1 M NaHCO_3 _(open orange symbol). **B**. The Δ*ebpR *mutant containing P_*ebpR*_::*lacZ *is represented by closed green diamond when grown in air and with open brown diamond when grown in the presence of 5% CO_2_/0.1 M NaHCO_3_.

As we previously noted [11], EbpR shares some homology with transcriptional regulators of the AtxA/Mga family. In this family, it has been shown that AtxA and Mga activate their regulon from mid-log to entry into stationary phase and that their regulon is affected by the presence of 5% CO_2_/0.1 M NaHCO_3 _[15,23]. We therefore tested the effect of CO_2_/NaHCO_3 _on *ebpR *and *ebpA *expression during growth using the P_*ebpR*_:: and P_*ebpA*_::*lacZ *fusions in OG1RF as shown in Fig. 2A. For the aerobic cultures, both *ebpR *and *ebpA *β-gal profiles followed the dome-shaped pattern over time, as described above. However, the presence of CO_2_/NaHCO_3 _led to a 2-3 fold increase in the β-gal units early during growth and, after the cultures entered stationary phase, *ebpR *and *ebpA *expression levels continued to increase for two hours and then showed only a slight decrease from 8 hr to 24 hr. At 24 hr, the β-gal units for OG1RF carrying the *ebpA *promoter were 13.9 in the presence of CO_2_/NaHCO_3 _compared to 0.4 aerobically, a 33-fold difference. Similarly, the β-gal units for OG1RF carrying the *ebpR *promoter were 1.2 in presence of CO_2_/NaHCO_3 _compared to 0.13 aerobically, a 9-fold difference.

To determine whether the CO_2_/NaHCO_3 _effect on *ebpA *expression was dependent on the presence of *ebpR*, we tested *ebpA *expression in an *ebpR *deletion mutant (TX5514). Using the *ebpR *deletion mutant (TX5514) containing P_*ebpA*_::*lacZ*, β-gal production was assessed in air and in the presence of 5% CO_2_/0.1 M NaHCO_3 _and β-gal production remained at the background level in both conditions (Fig. 2B). These results combined with our previously published results [11] indicate that, in air as well as in the presence of 5% CO_2_/0.1 M NaHCO_3_, *ebpR *is important for *ebpA *expression and that the 5% CO_2_/0.1 M NaHCO_3 _effect on *ebpA *expression level also requires the presence of *ebpR*.

**Figure 3:**
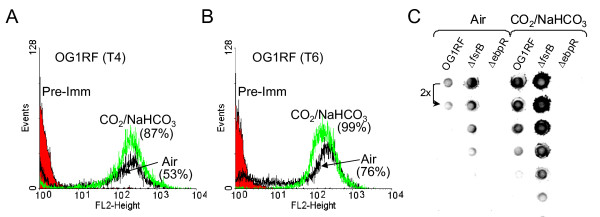
**Detection of EbpC produced by OG1RF, Δ*fsrB*, and Δ*ebpR*. A**. Flow cytometry analysis of OG1RF grown in air (black) or in the presence of 5% CO_2_/0.1 M NaHCO_3 _(green) labeled with an anti-EbpC rabbit polyclonal immune serum and detected with phycoerythrin. The cells were collected at "T4", which corresponds to the entry into stationary growth phase (4 hrs after starting the culture). The percentages between brackets indicate the percentage of positive cells (WinMDI 2.9, marker set for 500-1024). In red is represented OG1RF grown in air incubated with a pre-immune serum and detected with Phycoerythrin as negative control. **B**. Flow cytometry analysis was done in the same conditions as above with samples collected at "T6" which corresponds to early stationary growth phase. **C**. An equal amount (by BCA protein assay) of mutanolysin extract preparation was 2-fold serial diluted and spotted onto a nitrocellulose membrane. Pilus presence was detected with an anti-EbpC rabbit polyclonal immune serum.

We previously reported that only a fraction of the OG1RF cells were positive for pilus expression by immunofluorescence ([11]). To examine whether the presence of CO_2_/NaHCO_3 _affected the amount of pili per cell or the percentage of cells positive for pilus production, we used flow cytometry. As early as entry into stationary growth phase, a difference in the percentage of pilus positive cell was visible (Fig. 3A) with 53% positive when grown in air compared to 87% positive when grown in the presence of CO_2_/NaHCO_3_. The difference in the percentage of positive cells remained in later stages of growth. Specifically, Fig. 3B shows that, at 6 hr, 76% of the cells were positive when grown in air compared to 99% when the cells were grown in the presence of CO_2_/NaHCO_3_. The mean fluorescence intensity, between growth conditions and growth phases, remained constant with an average of 268. We also used anti-EbpC antibodies to probe mutanolysin extracts spotted on a dot blot for pilus production. An approximately four-fold increased signal density was observed in cells grown in the presence of CO_2_/NaHCO_3 _compared to the cells grown in air (Fig. 3C). Additionally, no signal was detectable under either growth condition in the mutant lacking *ebpR*, confirming the importance of *ebpR *for *ebpABC *expression and pilus production aerobically as well as in the presence of 5% CO_2_/0.1 M NaHCO_3_.

### The Fsr system effect on the *ebp *locus

**Figure 4:**
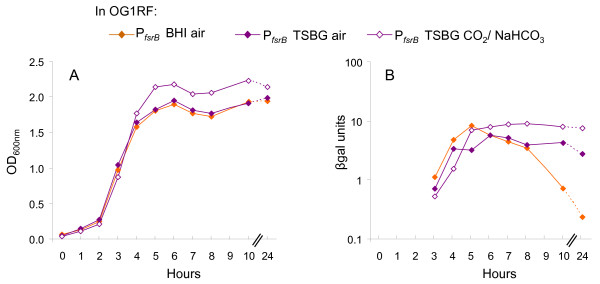
***fsrB *expression profile in OG1RF**. For β-gal assays, samples were collected every hour from 3 to 8 hr, then at 10 and 24 hr after starting the culture (x axis). All sets of cultures presented were analyzed concurrently. The figure is a representative of at least two experiments. The growth curves are represented in brown for cells grown in BHI-air and purple for cells grown in TSBG (thin line when grown in air, dense line when grown in the presence of 5% CO_2_/0.1 M NaHCO_3_). OG1RF containing P_*fsrB*_::*lacZ *was grown in BHI air (brown closed diamond), in TSBG- air (purple closed diamond) or in TSBG-5% CO_2_/0.1 M NaHCO_3 _(purple open diamond). A. OD_600 nm _readings. B. β-gal assays (β-gal units = OD_420 nm_/protein concentration in mg/ml).

We previously presented data in our microarray study suggesting that Fsr repressed the *ebpR-ebpABC *locus. However, the Fsr effect was only seen at one time point (during late log growth phase) using BHI grown cells [8]; in this medium, *fsrB *expression increased from mid-log to entry into stationary phase and then decreased rapidly [6]. Since our current study used mainly TSBG (our biofilm medium) as growth medium, we investigated the *fsrB *expression profile in TSBG. *fsrB *expression also increased until entry into stationary growth phase, reaching 66% of the expression detected in BHI broth, but then remained relatively constant throughout stationary phase (Fig. 4). These results indicate that *fsr *expression is variable in different conditions.

**Figure 5:**
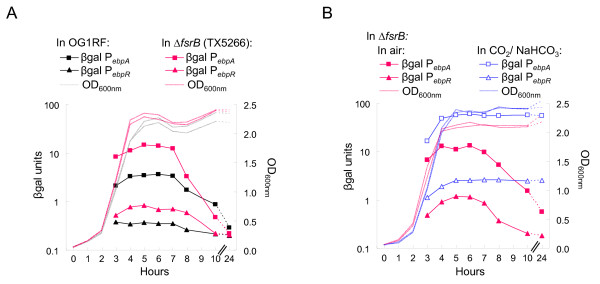
***ebpR *and *ebpA *expression profiles in TX5266 (Δ*fsrB *mutant)**. For β-gal assays, samples were collected every hour from 3 to 8 hr, then at 10 and 24 hr after starting the culture (x axis). The left axis represents the β-gal units (OD_420nm_/protein concentration in mg/ml). The right axis indicates the OD_600 nm _readings. All sets of cultures presented were analyzed concurrently. Each figure is a representative of at least three experiments. **A**. OG1RF containing either P_*ebpR*_::*lacZ *(black triangle) or P_*ebpA*_::*lacZ *(black square) and Δ*fsrB *containing either P_*ebpR*_::*lacZ *(pink triangle) or P_*ebpA*_::*lacZ *(pink square) were grown in TSBG aerobically. **B**. The Δ*fsrB *mutant (TX5266) containing either P_*ebpR*_::*lacZ *(triangle) or P_*ebpA*_::*lacZ *(square) was grown in TSBG aerobically (pink closed symbol) or in the presence of 5% CO_2_/0.1 M NaHCO_3 _(open blue symbol).

We next tested *ebpR *and *ebpA *expression using the P_*ebpR*_:: and P_*ebpA*_::*lacZ *fusions in OG1RF and TX5266 (Δ*fsrB *mutant), grown in parallel in TSBG aerobically. Both *ebpR *and *ebpA *gene expression profiles followed the same pattern in TX5266 as they did in OG1RF with an increase in expression until the culture reached stationary phase followed by a slow decrease (Fig. 5A). However, *ebpR *expression was 2-fold lower in OG1RF with 0.3 β-gal units compared to 0.8 β-gal units in TX5266 at entry into stationary phase. Similarly, *ebpA *expression was 4-fold lower in OG1RF with 3.7 β-gal units compared to 14.1 β-gal units in TX5266 early in stationary phase. These results confirm the role of the Fsr system as a repressor of the *ebpR-ebpABC *locus in TSBG, adding to the results obtained by microarray at one specific growth phase using cells grown in BHI.

To determine whether the CO_2_/NaHCO_3 _effect on *ebpA *and *ebpR *expression is mediated through Fsr, we looked at *ebpR *and *ebpA *expression in TX5266 in air and in the presence of 5% CO_2_/0.1 M NaHCO_3_. As shown in Fig. 5B, the *ebpA *and *ebpR *expression profiles in TX5266 grown aerobically and in the presence of 5% CO_2_/0.1 M NaHCO_3 _presented the same general profile as in OG1RF (Fig. 2A). That is, *ebpA *expression increased from 6.8 β-gal units at mid-log growth phase to 13.8 β-gal units at late log growth phase and decreased gradually to 0.6 β-gal units by 24 hr (late stationary). In the presence of 5% CO_2_/0.1 M NaHCO_3_, *ebpA *expression increased from 16.8 β-gal units at mid-log growth phase to 56.5 β-gal units (5-fold more than with cultures grown in air) at 6 hr and remained stable with 55.3 β-gal units at 24 hr. *ebpR *expression profile in TX5266 also remained higher in the presence of 5% CO_2_/0.1 M NaHCO_3 _vs. in aerobic conditions with 0.2 and 2.6 β-gal units, respectively, at 24 hr. Finally, we also examined the effect of CO_2_/NaHCO_3 _on *fsrB *expression by transferring the P_*fsrB*_::*lacZ *fusion into OG1RF and followed expression in air and in the presence of CO_2_/NaHCO_3_. In those conditions, *fsrB *expression was not significantly affected by the presence of CO_2_/NaHCO_3 _(Fig. 4). Our observation of a further increase in *ebpR *and *ebpA *expression in TX5266 in the presence of CO_2_/NaHCO_3 _as was observed in OG1RF (Fig. 2A and 5B), together with the lack of an effect of CO_2_/NaHCO_3 _on *fsr *expression, indicate that HCO_3_^- ^is not stimulating *ebpR *and *ebpA *expression via an effect on the Fsr system.

Finally, at the protein level, pilus production from the Δ*fsrB *mutant was compared with that of OG1RF. Cells were grown in TSBG aerobically or in presence of 5% CO_2_/0.1 M NaHCO_3_, and collected at 7 hr (stationary phase). As shown in Fig. 3C, a 3-5 fold increase in pilus production was observed in the Δ*fsrB *mutant compared to OG1RF with cells grown aerobically or in presence of 5% CO_2_/0.1 M NaHCO_3_. Similarly, 3-5 fold increase in pilus production was also seen with cells grown in the presence of 5% CO_2_/0.1 M NaHCO_3 _versus cells grown aerobically for both OG1RF and the Δ*fsrB *mutant. In conclusion, the differences observed in *ebp *mRNA expression levels between OG1RF and the Δ*fsrB *mutant and between the conditions used in this study (growth in air versus in the presence of 5% CO_2_/0.1 M NaHCO_3_) translated into comparable variations in pilus production at the surface of the cells.

### *ebpR *threshold level

**Figure 6:**
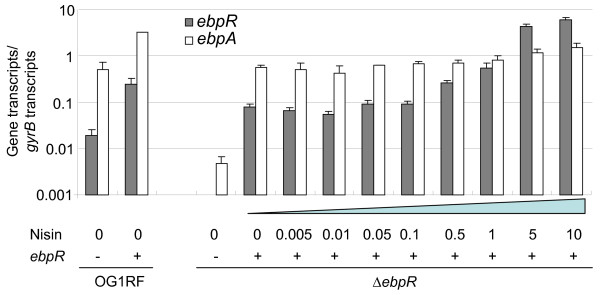
**Effect of nisin induction on *ebpR *and *ebpA *expression**. Cells were grown to an OD_600 nm _of ~0.8 (3 hr, late log exponential growth phase) and at this point cells were left untreated (0) or treated with increasing concentration of nisin (from 0.005 to 10 ng/ml). Then, cells were collected and RNA extracted. After reverse transcription, *ebpA *and *ebpR *cDNA was quantified by real time PCR. The strains were OG1RF or Δ*ebpR *(TX5514) carrying either the empty plasmid (-) or *ebpR *in trans under the nisin promoter (+). *ebpR *(gray bars) and *ebpA *(white bars) transcript levels were normalized with *gyrB *transcript levels. The data correspond to the mean of two independent experiments.

In the results obtained above, the *ebpR *and *ebpA *steady-state mRNA levels followed a similar pattern with *ebpA *expression being 7- to 37-fold higher than *ebpR *expression, depending on the technique. To investigate whether *ebpA *expression was directly related to the *ebpR *expression level, we introduced our previously cloned *ebpR *under a nisin inducible promoter (pTEX5515) into wild type OG1RF and into its Δ*ebpR *mutant, TX5514 [11]. Our previous experiments showed that, even without nisin induction, pilus production was detected at the surface of the cells of the *ebpR*-complemented Δ*ebpR *mutant, but not when the *ebpR *mutant carried the empty plasmid [11]. In this study, we investigated the steady-state mRNA level of *ebpR *and *ebpA *in different constructs with or without increasing amounts of nisin, compared to their respective levels in OG1RF carrying the empty vector, using qRT-PCR. The *ebpR *expression level in the *ebpR*-complemented Δ*ebpR *mutant was 0.08 (normalized to the *gyrB *expression level) without induction, increased 4-fold with 0.5 ng/ml nisin to 0.26 and reached 9.33 with 10 ng/ml nisin (Fig. 6), representing a 65-fold increase from 0 to 10 ng/ml nisin. In the same background, *ebpA *steady-state mRNA levels were only slightly affected with a basal expression level without nisin of 0.6 up to 1.5 with 10 ng/ml nisin (Fig. 6), a less than a 3-fold increase. However, as expected from our previous results, *ebpA *expression was 100-fold lower in the Δ*ebpR *mutant carrying the empty vector than in OG1RF carrying the empty vector or in the *ebpR*-complemented Δ*ebpR *mutant. We conclude from these experiments that, above the *ebpR *expression level provided by *ebpR *copy on pTEX5515 without induction, there is not a strong direct relationship between *ebpR *expression and *ebpA *expression.

### Bicarbonate effect on *ebpA *expression

**Figure 7:**
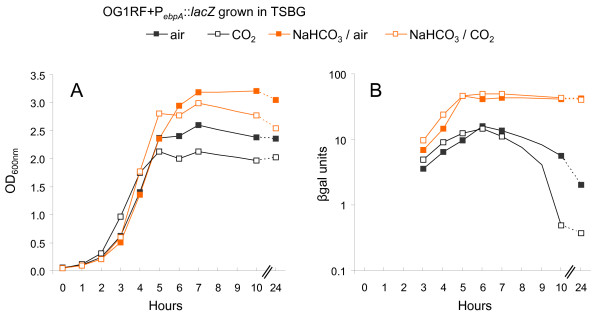
***ebpA *expression affected by NaHCO_3_, and not CO_2_**. For β-gal assays, samples were collected every hour from 3 to 8 hr, then at 10 and 24 hr after starting the culture (x axis). Growth curves of OG1RF containing P_*ebpA*_::*lacZ *are shown in air with a thin gray line, in NaHCO_3_/air with thin orange line, in CO_2 _with a dense gray line, and in NaHCO_3_/CO_2 _with a dense orange line. The β-gal assays for OG1RF containing P_*ebpA*_::*lacZ *are represented with closed black square, closed orange square, open black square, and open orange square when the cells were grown in air, 5% CO_2_, NaHCO_3_-air, and NaHCO_3_-5% CO_2_, respectively. All sets of cultures presented were analyzed concurrently. This figure is a representative of at least two experiments. A. OD_600 nm _readings. B. β-gal assays (β-gal units = OD_420 nm_/protein concentration in mg/ml).

Studies using *H. pylori *have shown independent effects of pH, CO_2_, and bicarbonate on gene expression (these three environmental elements being interconnected in vivo) where pH appears to be responsible for *H. pylori *orientation [24]. In contrast, bicarbonate and not CO_2 _appears to be the inducer of expression of the *B. anthracis *toxins [25]. Using the P_*ebpA*_*::lacZ *fusion in OG1RF, we first investigated the independent effect of CO_2 _and NaHCO_3 _on *ebpA *in buffered TSBG with or without the presence of 0.1 M NaHCO_3 _and/or 5% CO_2_. pH was controlled during the experiment and remained at pH 7.5 ± 0.25. As shown in Fig. 7, *ebpA *expression in TSBG-air did not differ appreciably from that in TSBG- 5% CO_2_, reaching a peak of expression early in stationary phase (15.8 and 14.5 β-gal units, respectively); expression then decreased to 2 and 0.4 β-gal units, respectively, at 24 hr. In the presence of NaHCO_3_, *ebpA *expression peak was ~4-fold higher with 46.5 β-gal units for the NaHCO_3_-air culture at entry into stationary phase (5 hr) compared to 9.8 β-gal when the cells were grown without NaHCO_3_, and 46.0 β-gal units for the 5% CO_2 _plus NaHCO_3 _culture compared to 12.5 β-gal when grown in presence of CO_2 _only. The bicarbonate effect persisted late into stationary phase with 42.5 and 40.7 β-gal units when grown in air-NaHCO_3 _and CO_2_-NaHCO_3 _respectively. A similar profile with increased *ebpR *expression in the presence of bicarbonate but not in presence of CO_2 _was also observed (data not shown). Furthermore, the differential effect of CO_2 _and NaHCO_3 _was also detected in BHI or when potassium bicarbonate was used as a source for HCO_3_^- ^(data not shown). Taken together, these results demonstrate that the increase in *ebpR *and *ebpA *expression is caused by the addition of HCO_3_^- ^and not CO_2_.

**Figure 8:**
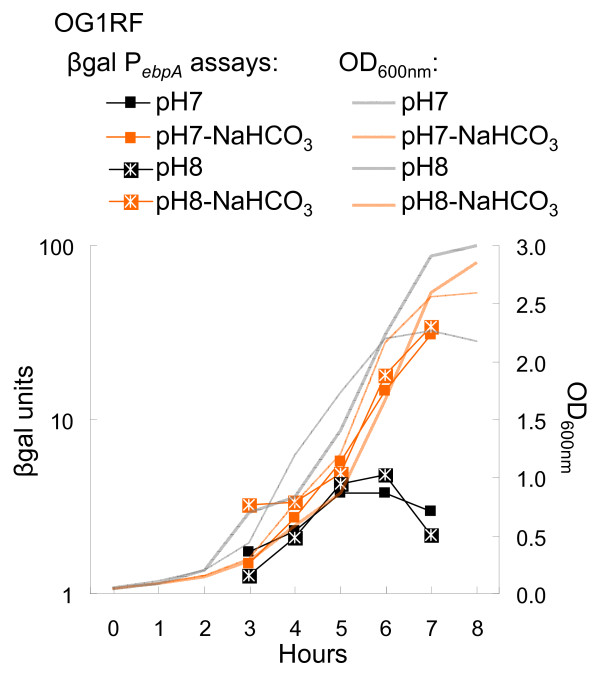
**pH and NaHCO_3 _effect on *ebpA *expression**. OG1RF containing P_*ebpA*_::*lacZ *was used in these experiments. Growth curves are represented in thin gray line for pH 7 aerobically, thin orange line for pH 7-Air/NaHCO_3_, dense gray line for pH 8 aerobically, and dense orange line for pH 8-Air/NaHCO_3_. All sets of cultures presented were analyzed concurrently. This figure is a representative of at least two experiments. A. OD_600 nm _readings. B. β-gal assays (β-gal units = OD_420 nm_/protein concentration in mg/ml).

Since NaHCO_3 _is in equilibrium with H_2_CO_3_, HCO_3_-, and CO_3_^2- ^depending of the pH, temperature and partial pressure of CO_2_, we next tested a possible pH effect on *ebpA *expression when cells were grown in buffered TSBG. In a preliminary experiment, OG1RF (P_*ebpA*_::*lacZ*) was grown in buffered TSBG with pH ranging from 5 to 9. Severe growth inhibition was observed at pH 5 and 9 with mild growth inhibition at pH 6, compared to unaffected growth at pH 7 and 8 (data not shown). Consequently, further experiments were conducted with buffered media with pH 7 and 8 only. Without the addition of sodium bicarbonate, *ebpA *expression levels of cells grown at pH 8 ± 0.25 were comparable with the levels in cells grown at pH 7 ± 0.25 (Fig. 8). However, adding NaHCO_3 _led to a 4- to 5-fold increase in β-gal production at either pH (pH was controlled during the experiment and remained constant with a ± 0.25 variation). For example, β-gal units were 9.4 at 6 hr for cells grown at pH 7-air, while at the same time point and pH, β-gal units were 40.1 when grown in the presence of NaHCO_3_. In conclusion, between pH (range 7-8), CO_2 _and bicarbonate, bicarbonate appears to be the main environmental inducer of the *ebpABC *operon.

### Effect of bicarbonate exposure on the OG1RF transcriptome

In an effort to begin to delineate the "bicarbonate regulon", we used microarray analysis with cells grown to late exponential growth phase (3 hr) and then submitted to a 15 min exposure with 0.1 M NaHCO_3_. Our goal was to define the first set of genes affected by the presence of bicarbonate. Out of the 73 genes that were differentially expressed (abs(fold)>2, *P *< 0.05, data deposited at ArrayExpress, additional file 1), only two genes were repressed by the presence of bicarbonate more than 5-fold (EF0082 and EF0083 with 9.9- and 7-fold, respectively) while four genes were activated more than 5-fold (EF0411-3 with ~10-fold, and EF2642 with 6.5-fold). EF0082 is part of the *ers *regulon (*ers *encodes a PrfA-like protein involved in the *E. faecalis *stress response [26,27]), but its function remains unknown, as is also true for EF0083. The EF0411-3 genes appear to be organized as an operon and encode proteins with the characteristics of a mannitol PTS system. EF2642 also appeared to be expressed in an operon with EF2641, which was also activated (4.1-fold, *P *< 0.05). EF2641 and EF2642 encode a putative glycine betaine/L-proline ABC transporter ATP-binding protein and permease protein, respectively. Those results were confirmed by qRT-PCR with a decrease of 32-fold for EF0082 in the presence of bicarbonate while EF0411 and EF2641 expression levels increased in the presence of bicarbonate by 24-fold and 8.5-fold, respectively (results not shown). The *ebpR-ebpABC *locus did not appear to be affected in these conditions (late log growth phase following a 15 min. incubation time with 0.1 M NaHCO_3_), suggesting that the bicarbonate effect on the *ebpR-ebpABC *locus may be indirect, requiring a cascade of events.

## Discussion

We previously noted that EbpR shares homology with the AtxA/Mga family [11]. Regulators in this family have been shown to be active toward their target(s) in the presence of CO_2 _or CO_2_/HCO_3_^-^. While *atxA *is constitutively expressed, *acpA *and *acpB *(also members of the AtxA/Mga family) as well as *mga *are activated by the presence of CO_2_. In the work described here, we present evidence that bicarbonate is a strong inducer of the *ebpR-ebpABC *locus and consequently of pilus presence. Among the other environmental conditions tested, pH appears to have a weak effect in the limited conditions tested, while CO_2 _had no effect. Although *ebpR *and *ebpA *expression levels share a similar pattern, we were not able to show that an increase in *ebpR *expression, beyond a certain level, resulted in a proportional further increase of *ebpA *expression. Finally, the Fsr system affects expression of the *ebpR-ebpABC *locus independently of either the growth phase or the presence of bicarbonate.

It is interesting that *ebpABC*, also shown to be important for *E. faecalis *virulence, responded to bicarbonate. Bicarbonate influences expression of *adcA *(encoding an adhesin [28]) and *kfc *(encoding a factor important for gut colonization) in *C. rodentium*, which are controlled by the bicarbonate regulator RegA [19], as well as the three toxin genes in *B. anthracis *[25]. Bicarbonate-mediated transcriptional activation may be a system to sense a change in the environment. For example, the proximal portion of the duodenum is exposed to intermittent pulses of gastric H(+) discharged by the stomach. To protect the epithelial surface, at least two HCO_3_^-^/Cl^- ^anion exchangers have been described as being responsible for the release of HCO_3_^- ^into the duodenum lumen [29]. We postulate that *E. faecalis *may be sensing this signal and consequently produces adhesin structures like the *ebpABC*-encoded pili to favor colonization of the intestinal track, similar to *adcA *in *C. rodentium*, the expression of which is controlled by bicarbonate and whose gene product has been shown to be involved in adherence to mammalian cells [28].

From the various results obtained in this study where expression of *ebpA *followed the same expression profile as the *ebpR *expression, we postulated that the *ebpA *expression level was proportionally linked to the *ebpR *expression. To investigate our hypothesis, we used an *ebpR *construct under the control of a nisin regulated promoter. However, as shown in Fig. 6, the *ebpR *expression level was already 2-fold higher in the complemented Δ*ebpR *strain (in the absence of nisin) when compared to its native level in wild type OG1RF (0.06 vs. 0.03) and was not detected (with a detection level of 10^-5 ^the level of *gyrB*) in the *ebpR *deletion mutant with the empty plasmid. We did not observe a strong effect on *ebpA *expression after nisin induction, leading to the conclusion that *ebpR *expression was already above the threshold required to significantly increase *ebpA *expression. We tried another construct pCJK96 (rhamnose induction [30]), but faced the same issues (data not shown). Thus, although we did not determine the threshold necessary for the *ebpA *expression, the presence of *ebpR *was confirmed to be critical for *ebpA *expression.

One difference between *ebpR *and *ebpA *expression profiles in the presence of bicarbonate (vs. absence of bicarbonate) occurred after entry into stationary phase. *ebpR *and *ebpA *expression without bicarbonate begins to decrease, while it remained constant in the presence of bicarbonate. This difference may be explained either by an induction pathway that remains active (in the presence of HCO_3_^-^) in stationary phase or by inhibition early in stationary phase of a repression pathway (e.g., quorum sensing or phase dependent regulator). The first mechanism would also explain the slight difference observed in the presence of HCO_3_^- ^during log growth phase. A potential candidate is a RegA homologue, an AraC/XylS-like regulator from *C. rodentium *[19]. Among the *E. faecalis *AraC/XylS-like regulators, none shares additional significant similarity with RegA. A second possibility would be a quorum sensing mechanism. A likely candidate would be the Fsr system [6]. However, the Fsr system, although a weak repressor of *ebpR*, does not appear to mediate the bicarbonate effect, since a similar *ebpA *expression pattern compared to OG1RF was observed in an *fsrB *mutant in the presence or absence of bicarbonate. Finally, we looked at the stress response pathway including *ers *and its regulon [26,27]. Interestingly, several members of the *ers *regulon were affected by a 15 min bicarbonate exposure, including EF0082-3 and EF0104-6. However, although both operons are activated by *ers*, EF0082-3 were strongly repressed (-8 fold), while EF0104-6 were activated (3 fold) by bicarbonate exposure. In addition, *ers *was not affected. In conclusion, the regulation pathways in *E. faecalis *resemble a network with several targets genes being under the control of independent regulation pathways illustrated by *ebpR-ebpABC *being independently a member of the bicarbonate and the *fsr *regulon, and EF0082 a member of the bicarbonate and *ers *regulon.

We also showed using microarray profiling that expression of many other genes (mostly PTS systems and ABC transporters) was altered in response to HCO_3_^-^. Among those genes are EF2641 and EF2642, which encode a putative glycine betaine/L-proline ABC transporter and permease protein, respectively. Interestingly, this ABC transporter shares some homology with the bicarbonate transporter described in *B. anthracis *(Tau family of ABC transporters) [25]. However, we did not find a TauA motif, that has been proposed as the bicarbonate binding motif, associated with the EF2641-2 locus or in available *E. faecalis *genomes including OG1RF. Interestingly, expression of *ebpR-ebpABC *was not affected by the 15 minutes bicarbonate exposure. Those results could be explained by the need of a cascade of events for a bicarbonate effect on *ebpR-ebpABC *expression or that the cells need an unknown factor, not present at the growth phase tested. Indeed, as seen in Fig. 2, Fig. 7, and Fig. 8, the greatest difference in *ebpR-ebpABC *expression was observed from mid stationary to late stationary growth phases (conditions that we found unsuited for microarray due to low and unstable mRNA expression). In conclusion, although we did not detect an effect of 15 minutes bicarbonate exposure on *ebpR-ebpABC *by microarray, the bicarbonate regulon was shown to share some components with the *ers *regulon and a later bicarbonate effect on *ebp *expression was shown by β-gal assays, qRT-PCR and western blot.

Finally, we have previously shown in the rat endocarditis model that an *fsrB *mutant is less attenuated than a *gelE *mutant [31]. Since, in the absence of the Fsr system, weak transcription of *gelE *was detected, it was postulated that the increase in virulence of the *fsrB *mutant compared to the *gelE *mutant might be a consequence of the residual production of gelatinase. However, since pilus production is also important in the rat endocarditis model [9], we can now postulate that, in the absence of the Fsr system as well as in presence of bicarbonate (by far the most important buffer for maintaining acid-base balance in the blood), pilus production increases, potentially causing the increased virulence of the *fsrB *mutant compared to the *gelE *mutant.

## Conclusion

Considering that bicarbonate is an activator of the *ebpR-ebpABC *locus and that this locus is ubiquitous among *E. faecalis *isolates (animal, commensal, and clinical isolates) [9], these results seem to suggest an intrinsic aptitude of this species for pilus production which could play an important role in colonization of both commensal and pathogenic niches. Future studies should assess expression of the *ebpR-ebpABC *locus and the role of pili in a gut colonization model.

## Methods

### Strains, media, growth conditions

The strains used in this study are listed in Table 1. All strains were routinely grown in brain heart infusion broth (BHI broth; Difco Laboratories, Detroit, Mich.) at 150-200 rpm aerobically or on BHI agar at 37°C, unless otherwise indicated. Tryptic soy broth (Difco Laboratories, Detroit, Mich.) with 0.25% glucose (TSBG) was used to test strains for biofilm production, one of the assays where both *ebpR *and *ebpA *mutants are attenuated compared to OG1RF [9,11].

**Table 1:** Strains and plasmids used in this study

Strain or Plasmid	Relevant characteristics	Source or reference
*E. coli *strains		
TG1	*E. coli *general cloning host	[35]
*E. faecalis *strains		
OG1RF	*E. faecalis*. Fus^R^, Rif^R^	[36]
TX5266	OG1RF *fsrB *deletion mutant, deletion from bp 79 to 684 of *fsrB*. Fus^R^, Rif^R^	[6]
TX5514	OG1RF *ebpR *deletion mutant, deletion from -5 bp to +1337 bp of *ebpR*. Fus^R^, Rif^R^	[11]
TX5584	TX5514(pMSP3535). Erm^R^, Fus^R^, Rif^R^	[11]
TX5582	TX5514(pTEX5515); *ebpR *mutant containing *ebpR *gene cloned into pMSP3535. Erm^R^, Fus^R ^, Rif^R^	[11]
TX5583	OG1RF(pMSP3535). Erm^R^, Fus^R^, Rif^R^	This study
TX5581	OG1RF(pTEX5515); *ebpR *mutant containing *ebpR *gene cloned into pMSP3535. Erm^R^, Fus^R ^, Rif^R^	This study
Plasmids		
pTCV-*lacZ*	Shuttle vector containing promoterless *lacZ*. Erm^R^	[32]
pMSP3535	Nisin inducible expression shuttle vector with pAM*β*1 and ColE1 replicons. Erm^R^	[37]
pTEX5269	*fsrB *promoter cloned upstream of *lacZ *in pTCV-*lacZ *(P_*fsrB*_::*lacZ*), from bp -110 to -8 (103 bp) relative to *fsrB *start codon; Erm^R^	[6]
pTEX5585	*ebpA *promoter cloned upstream of *lacZ *in pTCV-*lacZ *(P_*ebpA*_::*lacZ*), from -221 bp to +80 bp (301 bp) relative to *ebpA *start codon. Erm^R^	This study
pTEX5586	*ebpR *promoter cloned upstream of *lacZ *in pTCV-*lacZ *(P_*ebpR*_::*lacZ*), from -248 to + 53 bp (301 bp) relative to *ebpR *start codon. Erm^R^	[11]
pTEX5515	pMSP3535 with *ebpR *from -20 bp to +1561 bp from the ATG. This *ebpR *fragment contains the full ORF and the RBS of *ebpR*. Erm^R^	[11]

For all assays, strains were first streaked on BHI agar with the appropriate antibiotics, as needed. Five to ten colonies were inoculated into BHI broth and grown overnight (with antibiotics when appropriate), then cells were diluted so that the starting optical density at 600 nm was 0.05. For cultures grown in the presence of bicarbonate, a solution of 9% sodium bicarbonate was freshly prepared, filtered, and added for a final concentration of 0.8% (0.1 M final). The cultures were buffered with 100 mM 4-(2-hydroxyethyl)-1-piperazineethanesulfonic acid (HEPES) for a final pH of 7.5 ± 0.25 or as indicated. For comparison between cultures grown with and without bicarbonate, an equal volume of water was added to the culture without added bicarbonate. The cultures were then placed on a rotating platform set at 150 rpm at 37°C aerobically or in a 5% CO_2 _atmosphere. The pH was monitored during growth and remained at 7.5 ± 0.25. For each set of results, the cultures and following assays were analyzed concurrently. The presence of none of the four *lacZ *constructs (P_*TCV*_, P_*ebpA*_, P_*ebpR*_, and P_*fsrB*_) affected the growth of their host (OG1RF, Δ*ebpR*, or Δ*fsr*) in the conditions tested. To obtain accurate readings, cultures from 3 hr to 24 hr were diluted 5-fold before determining the OD.

### Construction of the ef1091 promotor fusion

The same protocol was used to create the P_*ebpA*_::*lacZ *fusion as previously described for the P_*ebpR*_::*lacZ *fusion [11]. The primers c**gggatcc**aagactacgccgaaaacc (introduced restriction sites are highlighted in bold) and g**gaattc**acacgaatgatttcttcca were used to amplify from 221 bp upstream to 80 bp downstream of the *ebpA *start codon (301 bp total). The fragment was amplified by PCR, cloned into pGEM-T-Easy vector (Promega, Madison, WI), sequenced, and then subcloned into pTCV-*lacZ *[32] using EcoRI and BamHI sites. After transfer into OG1RF, TX5266 (Δ*fsrB*), and TX5514 (Δ*ebpR*), the plasmids were then purified and confirmed again by sequencing using previously published primers Vlac1 and Vlac2, which are located upstream and downstream of the promoter area [32].

### β-galactosidase assay

Assays were performed according to the protocol of Hancock *et al*. [33] with some modifications. Following growth in the designated culture conditions and at each time point mentioned, a sample was collected (~2 × 10^9 ^CFU), centrifuged, and the pellet frozen until used. Cell pellets were resuspended in 1 ml of 1/10 Z buffer (Z buffer: 60 mM Na_2_HPO_4_, 40 mM NaH_2_PO_4_, 10 mM KCl, 1 mM MgSO_4_, [pH 7.0]). The cell suspension was transferred to a 2.0-ml tube containing a 0.5 ml volume of 0.1 mm diameter zirconia beads (BioSpec Products, Bartlesville, Okla.). The cells were disrupted using a vortex adapter for 5 min, then centrifuged at 13.6 K rpm for 1 min. Serial dilution of the aqueous layer was used in a β-galactosidase assay as described by Miller [34] with a final volume of 200 μl (96-wells microtiter plate). Twenty-five μl were assayed for total protein using the BCA protein assay kit (Pierce, Rockford, IL). Due to day to day variability, only data obtained within the same experiment (with cultures grown and samples assayed in parallel) were used for comparisons. To normalize the samples assayed in parallel, we used the total protein content as described in [33]. Experiments were repeated on at least two independent occasions and β-gal units for each experiment corresponded to OD_420 nm_/protein concentration in mg/ml. The figures show data from one representative experiment.

### RNA purification for qRT-PCR

To follow gene expression in OG1RF during growth in TSBG at 37°C, 150 rpm, samples were collected every hour from three to 7 hr after starting the culture. For the nisin induction assay, cells were grown to an OD_600 nm _of ~0.8 (3 hr, late log exponential growth phase), and at this point cells were left untreated or treated with increasing concentration of nisin (from 0.005 ng/ml to 10 ng/ml). In each case, an equivalent of OD_600 nm _~ 1 of cells was centrifuged, and the pellet was conserved at -80°C. RNA and cDNA were prepared using the methods described before [8]. Quantitative PCR on cDNA was performed using SYBR green PCR master mix kit (Applied Biosystems, Foster City, CA) and a 7500 Real-Time PCR system (Applied Biosystems). *ebpA *was selected for those experiments because it is the first gene of the *ebpABC *operon. The following primers were used: *gyrB*, accaacaccgtgcaagcc and caagccaaaacaggtcgcc; *ebpA*, aaaaatgattcggctccagaa and tgccagattcgctctcaaag; *ebpR*, acggatatggcaaaaacg and agaagagcgactaatattgatgg; EF0082, aaactccttgaactgattgg and ccagataaagaatgcccata; EF0411, agctgaactaacggaacaag and tcttttaagagcgaaaccac; and EF2641, attcgtggtgttcctaaaga and catcccaccagataattgac. For each primer set, a reference curve was established using a known amount of gDNA purified from OG1RF. The amount (in ng/ml) obtained for the gene of interest transcripts were normalized with the amount of *gyrB *transcripts.

### Microarray analysis

The BHI cultures of OG1RF were started as described above. Cultures were grown to an OD_600 nm _of ~0.8 (3 hr, late log exponential growth phase), and at this point 25 ml of culture were centrifuged and resuspended in either BHI-buffered or BHI-buffered with 0.1 M bicarbonate, incubated for 15 min at 37°C @ 150 rpm, then centrifuged and the pellet conserved at -80°C until use. The microarray consists of 70-mer oligonucleotides that were printed on a GAPS II slide (Corning Incorporated, Corning, NY) at the University of Texas Medical School Microarray Core Laboratory. The RNA preparation, probe labeling, hybridization, data acquisition and statistical analysis were performed following the same methods as described previously [8]. The results of the bicarbonate induction are deposited at ArrayExpress http://www.ebi.ac.uk/microarray-as/ae/ under accession number E-MEXP-2518.

### Flow cytometry analysis

An equivalent of ~ 1 OD_600 nm _of culture was collected for flow cytometry analysis, centrifuged and the pellet frozen until used. The pellet was then washed twice with 1 ml of PBS (80 mM Na_2_HPO_4_, 20 mM NaH_2_PO_4_, 100 mM NaCl, pH 7.5), resuspended in 0.5 ml of paraformaldehyde buffer (4.4% w/v paraformaldehyde, 30 mM Na_2_HPO_4_, 30 mM NaH_2_PO_4_), and incubated at RT for 15 min. The cells were pelleted and resuspended in 0.5 ml of PBS-2% BSA, and subsequently placed at -80°C for at least an hour. Before labeling, the cells were washed twice in PBS. A pellet corresponding to 10^8 ^CFU was resuspended in 100 μl of PBS with the anti-EbpC polyclonal rabbit serum at a 1:1000 dilution, and incubated at 4°C for 2 h. After centrifugation and two washes with PBS, the cells were resuspended in 100 μl of PBS with R-Phycoerythrin-conjugated affinipure F(ab')_2 _goat anti-Rabbit IgG (H+L) (Jackson ImmunoResearch Laboratories, Inc) at a dilution of 1:100, and incubated at 4°C for 2 h. The cells were then washed twice, resuspended in 1 ml PBS, and conserved at 4°C until they were analyzed with a BD FACSCalibur™ system (BD Biosciences, San Jose, CA).

### Protein extraction and dot blot

Surface protein extracts from *E. faecalis *OG1RF and derivatives were prepared using mutanolysin (Sigma Chemical Co., St. Louis, MO). Cells grown at 37°C in specified conditions were collected at 7 hr after starting the culture. The cells were washed and resuspended in 1/100 volume of 0.02 M Tris-HCl (pH 7.0)-0.01 M MgSO_4 _buffer. Mutanolysin was added to a final concentration of 5 U for an equivalent of 1 OD_600 nm _of cells and incubated at 37°C for 1 hr. The supernatants were collected after centrifugation at 13.6 K rpm for 5 min. An equal amount of mutanolysin extract preparation (quantified using the BCA protein assay kit) was 2-fold serial diluted and was spotted onto NitroPure (GE Water and Process Tech., Watertown, MA) using the Bio-Dot^® ^Microfiltration Apparatus (Biorad, Hercules, CA). The membranes were incubated with anti-EbpC rabbit polyclonal antiserum [9] at a dilution of 1:2000, followed by protein A-horseradish peroxidase conjugate (1:5000). Pilus production was then revealed using chemiluminescence (Amersham, Piscataway, NJ).

## Authors' contributions

AB and BEM designed the study. AB performed the experiments except the beta-galactose assays done also by LCT. AB wrote the draft of the manuscript. BEM assisted in critical review of the manuscript. All authors read and approved the final manuscript.

## Supplementary Material

Additional file 1 **Microarray results following 15 minutes bicarbonate induction**. Define the first set of genes affected shortly after addition of bicarbonate to the medium. Click here for file
