# Supplementary figures and images for: Bicarbonate enhances expression of the endocarditis and biofilm associated pilus locus, ebpR-ebpABC, in Enterococcus faecalis
Source: BMC Microbiol. 2010 Jan 21;10:17. doi: 10.1186/1471-2180-10-17 (PMC2824692; doi:10.1186/1471-2180-10-17)

Supplemental table S1: Microarray results following 15 minutes bicarbonate induction.


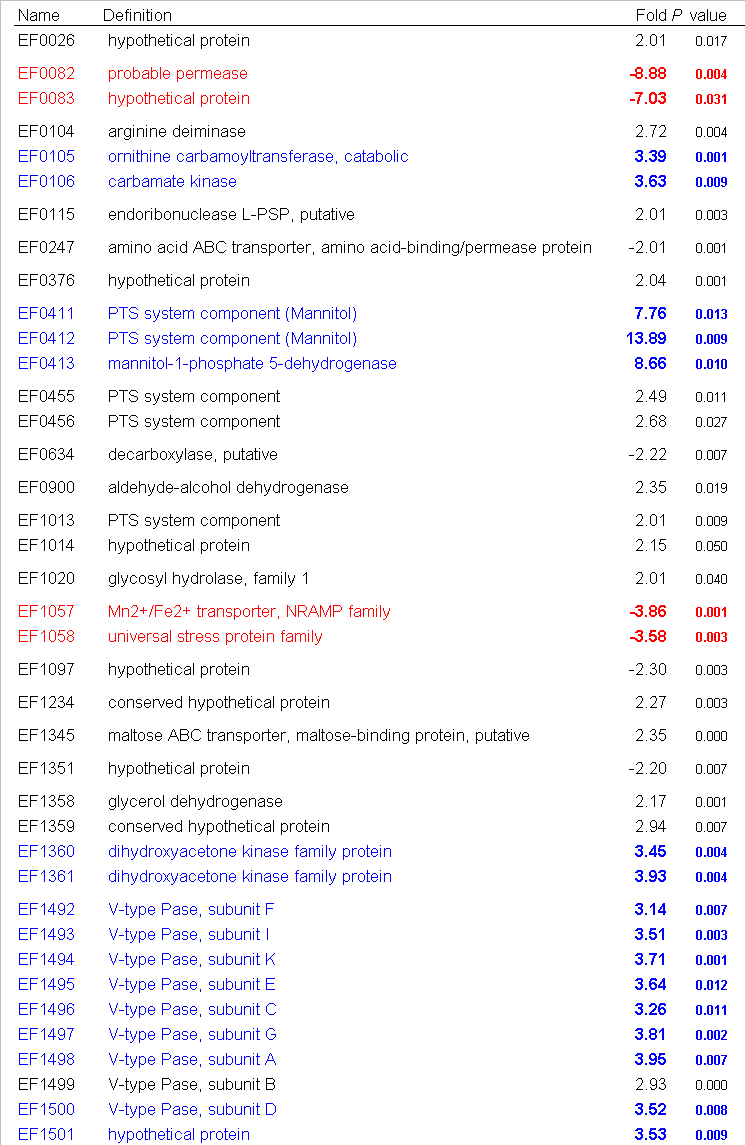


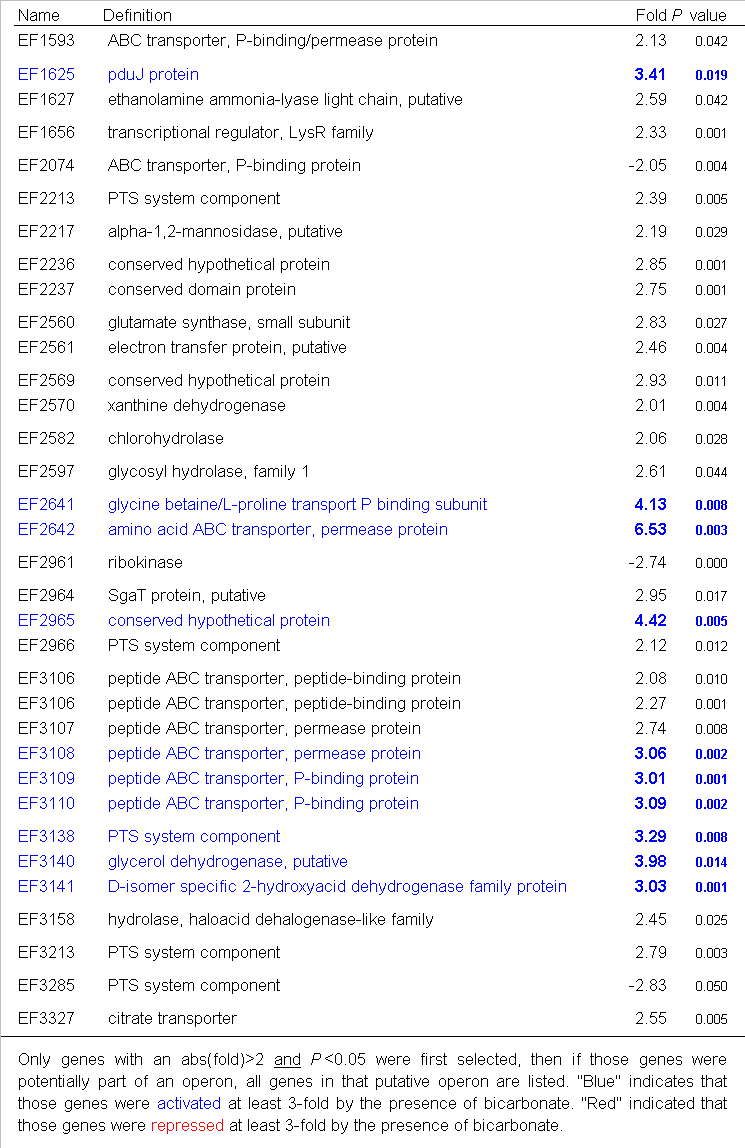

Supplement: Additional file 1 — Microarray results following 15 minutes bicarbonate induction. Define the first set of genes affected shortly after addition of bicarbonate to the medium. [file 1471-2180-10-17-S1.DOC]
